# Shrinking of Extracellular Space During Metabolic Stress Accelerates Amyloid-β Aggregation

**DOI:** 10.3390/biom16071053

**Published:** 2026-07-18

**Authors:** Laura F De Oliveira, Kanchana Karunarathne, Dalton Zona, Martin Muschol, Ghanim Ullah

**Affiliations:** Department of Physics, University of South Florida, Tampa, FL 33620, USA; lauraf2@usf.edu (L.F.D.O.); dilhanikanch@usf.edu (K.K.); dzona@usf.edu (D.Z.); mmuschol@usf.edu (M.M.)

**Keywords:** Amyloid β, aggregation, metablic stress, spreading depolarization, Alzheimer’s disease

## Abstract

Pathological states associated with metabolic stress, such as traumatic brain injury (TBI), hypoxia, ischemic stroke, and migraine, are considered elevated risk factors for developing Alzheimer’s disease (AD). However, the mechanism underlying the effect of these conditions on the progression of AD remains largely unknown. Here, we determine how metabolic stress associated with spreading depolarization (SD)—a hallmark of stroke, hypoxia, TBI, and migraine—modulates amyloid β (Aβ42) aggregation kinetics through dynamic changes in extracellular space (ECS). To achieve this, we used ThT fluorescence to determine how the formation of different Aβ42 aggregate species depends on Aβ42 concentrations. Based on this input, we build a multiscale computational framework that integrates volume regulation, including its dependence on neuronal ion homeostasis, and Aβ42 aggregation kinetics. Our model predicts that neuronal swelling during SD accelerates aggregation, where the impact of metabolic stress is highly dependent on the timing relative to aggregation progression and the initial monomer concentration. At low monomer concentrations, early SD events promote off-pathway oligomer formation, while at higher concentrations they rapidly drive fibril formation to saturation. In the absence of mature fibrils, recurrent metabolic stress events further amplify oligomer accumulation, whereas pre-existing fibril nuclei suppress oligomer formation at the expense of fibril nucleation and growth. Increasing the intensity of metabolic stress prolongs ECS shrinkage and enhances oligomer formation. These findings reveal a mechanistic link between SD-induced microenvironmental changes and Aβ aggregation dynamics, providing a quantitative framework for understanding how acute brain injury and metabolic stress may contribute to early AD pathogenesis.

## 1. Introduction

The onset of amyloid β (Aβ) oligomers and fibril formation is considered among the earliest molecular events in Alzheimer’s disease (AD) and the main cause of its pathogenesis [1,2,3]. In vivo, Aβ monomers are derived from the sequential cleavage of amyloid precursor protein (APP) by β- and γ- secretases [4,5,6] that occurs in a normal human brain. However, the conditions that initiate and promote the aberrant aggregation of Aβ, specifically in sporadic AD, are poorly understood.

In vitro and in vivo experiments have identified a myriad of Aβ aggregate species, ranging from small oligomers to curvilinear and long rigid fibrils (RFs), and established that Aβ aggregation proceeds via two parallel pathways [7,8,9]. The on-pathway leads to the formation of primary nuclei and their elongation into ordered curvilinear and rigid fibrils—species that are generally considered less acutely toxic but constitute the hallmark plaques of AD [10,11]. The off-pathway leads to the formation of globular oligomers (gOs)—metastable, soluble species that are now widely regarded as the primary neurotoxic agents in AD, capable of disrupting synaptic function and impairing membrane integrity [12,13,14,15]. The probability of off-pathway oligomer formation increases above a critical monomer concentration, creating a concentration-dependent competition between fibrillization and oligomerization [7,8,9]. Our previous work on Aβ dimer construct, lysozyme, Aβ40, and Aβ42 provides direct experimental and modeling support for this two-pathway framework [7,8,9,16], and a central goal of the present study is to determine how pathological conditions modulate the balance between these competing pathways.

The outcome of this on/off-pathway competition is governed by three key parameters: local Aβ monomer concentration, ionic strengths, and pH [7,9,17,18,19,20,21,22,23,24,25,26]. Under physiological conditions, these parameters are more likely to vary mildly since robust regulatory mechanisms prevent drastic changes, limiting the likelihood of conditions becoming favorable for aggregation. However, there are pathological brain states caused by metabolic stress and ionic homeostatic imbalance, where significant changes in all these three variables are observed simultaneously, which are acutely prone to aberrant aggregation.

The most prominent of these pathological states is spreading depolarization (SD). Metabolic stress and SD locally create aggregation-prone conditions by simultaneously shrinking the ECS, lowering pH, upregulating Aβ-cleaving enzyme (BACE1) activity, and reducing Aβ buffering [27,28,29,30,31]. SD is the electrophysiological event underlying several brain pathologies, including traumatic brain injury (TBI), migraine, aneurysmal subarachnoid hemorrhage, intracerebral hemorrhage, hypoxia, and ischemic stroke [32,33,34,35,36,37]. Remarkably, all these conditions are also considered elevated risk factors for developing AD [38,39,40,41]. For example, a history of TBI increases the risk of developing AD by 4.5 to 10 times [42,43,44,45,46] and reduces the age of AD onset by several years [47,48]. Furthermore, strong similarities exist in the regional neurodegeneration patterns associated with mild TBI and AD [49]. There is also evidence that TBI increases the risk of dementia in individuals with the E4 variant of the apolipoprotein E gene [50]—the largest known genetic risk factor for sporadic AD. Critically, the expression and activity of β-secretase BACE1—the rate-limiting enzyme in this cleavage process [4]—are upregulated 2–3-fold under hypoxic and ischemic conditions [29,31,38], providing a direct mechanistic link between metabolic stress and increased Aβ production.

SD is a depolarization wave sweeping the tissue that raises the neuronal membrane potential close to 0 mV. It results in massive ions and water displacements, abolishment of membrane ionic gradients, and a more than 50% drop in local ATP [27,28,33,35,51]. Typically, SD waves are associated with a significant rise in the extracellular K^+^ from ~3 mM to as high as ~60 mM [23]. Oxygen–glucose deprivation also leads to SD waves, which are accompanied by a rise in the extracellular K^+^. Similar dramatic changes occur in other extra- and intracellular ion concentrations, glutamate, and pH [35,52,53,54]. The rise in intracellular Na^+^ and Cl^−^ leads to substantial neuronal and astrocytic swelling [55], and consequently a shrinkage of the ECS from 18–22% to 5–9% of total tissue volume, hence quadrupling the concentrations of all extracellular ions and molecules, including Aβ. In addition, high extracellular K^+^ causes vasoconstriction that results in hypoxia [27,56,57,58], switching to anaerobic ATP production and accumulation of lactate. This leads to the failure of pH-regulating pumps and a drop in pH in the ECS to 6.1 near the ischemic core [52,59,60,61,62,63,64,65,66,67,68]. SD can also occur in clusters that persist for hours, and SD-like conditions may last for extended periods, as is usually observed in TBI and recurrent migraines [28,32,38]. Recurrent SD waves may therefore affect the growth kinetics of pre-existing Aβ aggregates in ways that compound over time.

In this paper, we focus on how neuronal swelling, and consequently shrinking of the ECS, affects the aggregation kinetics of Aβ42. We combine multiscale modeling with high-resolution microscopy to understand the onset and progression of different aggregate forms of Aβ42 in a normal brain during SD and related metabolic stress.

## 2. Methods

### 2.1. Experimental Methods: Oligomer vs. Fibril Formation as a Function of Aβ42 Concentration

#### 2.1.1. Aβ42 Solution Preparation

Lyophilized Aβ42 stock was acquired commercially (GL Biochem, Shanghai, China). The lyophilized peptides were dissolved at 1 mg/mL in 500 μL of 100 mM NaOH at pH 13 and injected into an FPLC (Akta Pure, Cytiva, Dreieich, Germany) with a Superdex 75 10/300 GL column (GE Healthcare, Chicago, IL, USA), equilibrated against a solution of 35 mM Na_2_HPO_4_ at pH 11. The monomer peak was collected and immediately stored on ice. Concentrations of the resulting Aβ42 monomer stock were determined by integrating the UV absorption trace from the FPLC using a molar absorptivity of ε_280_ = 1470 M^−1^ cm^−1^ [38,39]. The typical yield was 1.5 mL of Aβ42 at 40 μM to 80 μM. We note that quantification of Aβ42 using laboratory spectrofluorometers carries an estimated uncertainty of ~10–15% in absolute concentration due to the low absorptivity of the single Tyr residue at 280 nm. However, we integrated the high-resolution (mAU) signal of the FPLC UV detector to determine Aβ42 concentrations. In addition, all experiments used the same preparation batch and calibration, and relative concentration values—which govern the kinetic analysis—are more reliably determined. This approach is consistent with standard practice in the field [17,22].

#### 2.1.2. Aβ42 Aggregation Kinetics

A series of solutions at pH 7.4 and 150 mM NaCl with Aβ42 concentrations ranging from 2 μM to 50 μM was prepared. Low-binding 1.5 mL microcentrifuge tubes were filled with 500 μL of 35 mM Na_2_HPO_4_ buffer and 150 mM NaCl at pH 11. The purified monomer stock was diluted into these tubes. A small amount (<5 μL) of 1 M HEPES buffer at pH 4.5 was added to adjust the final solution pH to 7.4 right before plating. Thioflavin T (ThT) stock solutions (7.5 μL) were added for a final dye concentration of 15 μM. Three wells of a low-binding 96-well half-area microplate (#3881, Corning Life Sciences, Tewksbury, MA, USA) were filled with 150 μL of a given Aβ42/dye mixture. Plates were sealed with polyethylene sealing tape and incubated in a FLUOstar Omega fluorescence plate reader (BMG Labtech, Ortenberg, Germany) at 27 °C. The instrument was pre-equilibrated at 27 °C for at least 30 min before plating to ensure thermal stability. Temperature was maintained at 27 ± 0.3 °C throughout all kinetic measurements, as confirmed by the instrument’s internal thermocouple feedback system. ThT fluorescence was measured at 15 min. intervals (with 3 s of orbital shaking immediately prior to each fluorescence measurement; samples were otherwise stationary between reads—an intermittent shaking protocol standard for Aβ42 ThT kinetics assays [69] for 1–2 days.

ThT fluorescence was measured using a 448/10 nm excitation and 482/10 nm emission filter pair. Dye/buffer wells without protein served as a control for potential changes in dye fluorescence (bleaching, hydrolysis, etc.) unrelated to protein aggregation.

#### 2.1.3. Data Analysis

Dye traces were exported to Igor data analysis software (Igor 8.0, Wavemetrics, Lake Oswego, OR, USA) for further analysis. By dividing sample fluorescence values F(t) by their corresponding dye/buffer fluorescence at the initial measurement time F(B0), the raw fluorescence data were converted into fractional fluorescence enhancements [F(t)/F(B0)]. Normalized data were exported to Excel, and best-fit parameters extracted from fits to the numerical model described below.

### 2.2. Computational Methods

**Figure 1:**
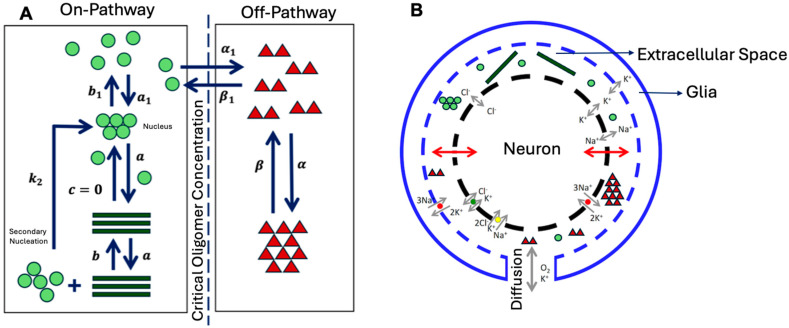
Schematic of the model. (**A**) A schematic of the aggregation kinetics of Aβ42. Along the on-pathway, in addition to primary nucleation, secondary nucleation contributes to RFs’ seed formation. Within the on-pathway, 5 monomers (individual spheres in light green) associate cooperatively in one step to form a nucleus (cluster of 5 spheres in light green). Beyond the nucleus, fibril growth ensues, continuing until all monomers are consumed, progressively increasing the size of RFs (rods in dark green). The irreversibility of RFs is indicated by the dissociation rate c = 0. The already existing RFs catalyze the formation of new ones through secondary nucleation with a rate constant k_2_. On the off-pathway, monomers first form the intermediate species (dimers shown as clusters of two triangles in red), followed by the final gOs species (cluster of 10 triangles in red). gOs are metastable, dissolving into monomers that eventually end up in RFs along the on-pathway. (**B**) A schematic of the neuronal model that allows the volume of the neuron to change as transmembrane osmolarity changes (red arrows), incorporating energy-dependent pumps on both neuron and glia (red bullets), and letting O2 and K diffuse with finite time to the cell (thick gray arrows). Transporters KCC2 (purple) and NKCC1 (yellow) regulate intracellular Cl^−^. Concentration of various Aβ42 species (same color coding as in panel (**A**)) changes as the ECS changes due to transmembrane osmolarity changes during metabolic stress.

The model integrates neuronal ion homeostasis and associated volume changes with the kinetics of Aβ42 aggregations in the ECS (Figure 1).

#### 2.2.1. Modeling Aβ42 Aggregation Kinetics

For simulating Aβ42 aggregation, ThT traces representing the formation of fibrils and oligomers were fitted by our previous model [7,9], which is a modified version of the model in [70]. A schematic of the model is shown in Figure 1A, where monomers can form on-pathway fibrils and off-pathway oligomers. The probability of fibrils and/or oligomer formation depends on the initial monomer concentration. Along the on-pathway, monomers ($\left[X_{1}\right]$) first form primary nuclei ([Y_n_], *n* = 5), followed by the growth into fibrils $\left[F^{\left(0\right)}\right]$. The concentration of monomers incorporated into the fibrils is represented by $\left[F^{\left(1\right)}\right]$. The off-pathway aggregates are formed at relatively higher monomer concentration, usually when the monomer concentration exceeds a critical value specific to each amyloidogenic species. Above this threshold, monomers can also form off-pathway globular oligomers via a two-step process. They initially form an intermediate dimer ($\left[Z_{k}\right]$), where k = 2, followed by aggregation into oligomers $\left[Z_{m}\right]$ where m represents the maximum number of monomers in the species (we use m = 10 in this work). We refer the reader to Refs. [7,9] for further details about the model.

In addition to changing the parameters to allow fitting the model to our new data, we change the rate equations such that they represent the rate at which the number of different species is changing instead of their concentrations. This change is necessitated by the fact that the volume of the ECS in our simulation is a dynamic variable, which changes as the extra- and intracellular ion concentrations change.

(1) $$\frac{dNX_{1}}{dt}=(-na_{1}{\left[X_{1}\right]}^{n}+{nb}_{1}\left[Y_{5}\right]-a\left[X_{1}\right]\left[F^{\left(0\right)}\right]+b\left[F^{\left(0\right)}\right]-k\alpha_{1}{\left[X_{1}\right]}^{k}+k\beta \left[Z_{k}\right]\;-\left(m-k\right)\alpha {\left[X_{1}\right]}^{\left(m-k\right)}\left[Z_{k}\right]+\left(m-k\right)\beta \left[Z_{m}\right]-nk_{2}{\left[X_{1}\right]}^{n}\left[F^{\left(1\right)}\right])\times {Vol}_{o},\;\frac{d{NY}_{n}}{dt}={(a}_{1}{\left[X_{1}\right]}^{n}-b_{1}\left[Y_{n}\right]-a\left[X_{1}\right]\left[Y_{n}\right]+k_{2}{\left[X_{1}\right]}^{n}\left[F^{\left(1\right)}\right])\times {Vol}_{o},\;\frac{d{NZ}_{k}}{dt}=\left(\left(\alpha_{1}{\left[X_{1}\right]}^{k}-\beta_{1}\left[Z_{k}\right]\right)-\left(\alpha {\left[X_{1}\right]}^{\left(m-k\right)}\left[Z_{k}\right]-\beta \left[Z_{m}\right]\right)\right)\times {Vol}_{o},\;\frac{d{NZ}_{m}}{dt}=\left(\alpha {\left[X_{1}\right]}^{\left(m-k\right)}\left[Z_{k}\right]-\beta \left[Z_{m}\right]\right)\times {Vol}_{o},\;\frac{d{NF}^{\left(0\right)}}{dt}=\left(a\left[X_{1}\right]\left[Y_{n}\right]\right)\times {Vol}_{o},\;\frac{d{NF}^{\left(1\right)}}{dt}=\left(\left(n+1\right)a\left[X_{1}\right]\left[Y_{n}\right]+a\left[X_{1}\right]\left[F^{\left(0\right)}\right]-b\left[F^{\left(0\right)}\right]\right)\times {Vol}_{o}.$$

where *N* in front of the species indicates their number concentrations. For example, *NX_1_* is the number of monomers, whereas [*X_1_*] is the concentration of monomers. ${Vol}_{o}$ is the volume of the ECS.

Association rate constants are denoted by $a,a_{1},\alpha ,\alpha_{1}$ while dissociation rate constants are given by $\beta ,\beta_{1},b,b_{1}$ (Table 1). As shown in the Results section, the parameter $k_{2}$ representing the secondary nucleation rate varies as a function of the initial monomer concentration and is formulated as

(2) $$k_{2}=3.082\times 10^{-9}{exp}^{\left(-0.2625[X_{1}]\right)}+1.5239\times {10^{-7}exp}^{\left(-0.73[X_{1}]\right)}.$$

in units of 1/(μM^5^.s).

**Figure 2:**
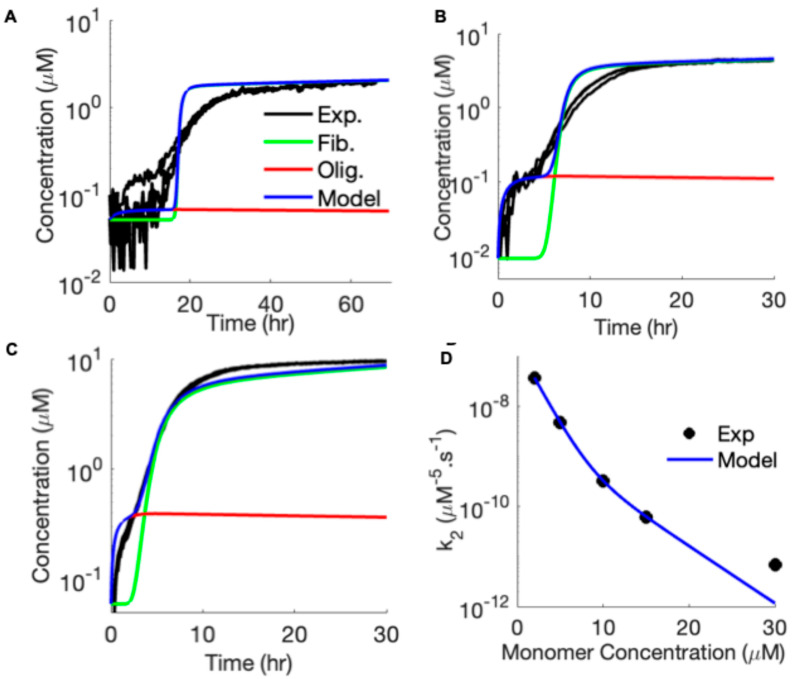
The model reproduces the observed transition from predominantly sigmoidal to biphasic growth as we increase initial monomer concentration. Sample fits to ThT signal at 2 μM (**A**), 5 μM (**B**), and 10 μM (**C**), where the black, red, green, and blue lines represent experimental data, off-pathway oligomers, on-pathway RFs, and the overall aggregated species, respectively. Experiments at each monomer concentration were repeated three times, as shown by the three black lines in each panel. (**D**) The rate of secondary nucleation as a function of monomer concentration obtained from model fits to experimental data similar to panels (**A**–**C**) (black) and its functional form (blue).

Equation (2) is an empirical two-phase decaying exponential fit to the secondary nucleation rate k_2_ values extracted from fitting the aggregation model to ThT kinetics data at different monomer concentrations (Figure 2D). The functional form captures the observed rapid initial decrease in k_2_ at low-to-intermediate $\left[X_{1}\right]$, followed by a slower secondary decline at higher concentrations. Physically, this behavior reflects the progressive inhibition of secondary nucleation as off-pathway oligomers—which form in increasing amounts at higher $\left[X_{1}\right]$—decorate the lateral surfaces of existing fibrils and block secondary nucleation sites [16]. The two-exponential form was selected over a single exponential as it provided a substantially better fit to the experimentally derived k_2_ values across the full concentration range tested.

#### 2.2.2. Neuronal Model

To model neuronal membrane potential, ion homeostasis, and volume regulation during SD and related metabolic stress events, we adopt the formalism developed in our previous work [71]. Briefly, the model describes a single-compartment neuron coupled to an ECS and surrounded by a simplified glial compartment. The neuron’s membrane potential is governed by voltage-gated Na^+^ and K^+^ currents (Hodgkin-Huxley formalism), leak currents, and active Na^+^/K^+^-ATPase pumps. Ion concentrations in the intra- and extracellular spaces are dynamic variables governed by conservation of charge and mass. The model includes glial K^+^ uptake, K^+^/Cl^−^ (KCC2), and Na^+^/K^+^/Cl^−^ (NKCC1) cotransporters that regulate intracellular Cl^−^, and oxygen-dependent pump rates that allow simulation of hypoxic/ischemic conditions. The volume of the neuron and ECS is dynamically updated based on osmotic gradients between compartments. The resulting neuronal swelling during SD—and the consequent ECS shrinkage—directly alters Aβ42 concentrations in the ECS, linking the neuronal model to the aggregation kinetics model.

#### 2.2.3. Modeling Neuronal Membrane Potential

The neuronal membrane potential (*V*) is modeled as

(3) $$C\frac{dV}{dt}=-I_{Na}-I_{K}-I_{Cl}-\frac{I_{pump}}{\gamma }.$$

The Na^+^, K^+^, and Cl^−^ currents are formulated by Hodgkin and Huxley type equations:

(4) $$I_{Na}=G_{Na}m^{3}h\left(V-E_{Na}\right)+G_{Na,L}\left(V-E_{Na}\right),\;I_{k}=G_{K}n^{4}\left(V-E_{K}\right)+G_{K,L}\left(V-E_{k}\right),\;I_{Cl}=G_{Cl,L}\left(V-E_{Cl}\right),$$

where $G_{x}$, $G_{x,L}$, $E_{x}$ represents the maximum conductance for ion *x*, the conductance of leak channels, and the reversal potential for ion *x*. h, m, and *n* are the gating variables of the ${Na}^{+}$ or K^+^ channels as in the original Hodgkin–Huxley formalism [72]:

(5) $$\frac{dq}{dt}=\alpha_{q}\left(1-q\right)-\beta_{q}q,q=m,h,n.$$

Here, the voltage-dependent forward ($\alpha_{q}$) and backward ($\beta_{q})$ rates are given as

(6) $$\alpha_{m}=\frac{0.32\left(V+54\right)}{1-\exp\left(-\frac{V+54}{4}\right)},\;\beta_{m}=\frac{0.28\left(V+27\right)}{\exp\left(\frac{V+27}{5}\right)-1},\;\alpha_{h}=0.128\exp\left(-\frac{V+50}{18}\right),\;\beta_{h}=\frac{4}{1+\exp\left(-\frac{V+27}{5}\right)},\;\alpha_{n}=\frac{0.032\left(V+52\right)}{1-\exp\left(-\frac{V+52}{5}\right)},\;\beta_{n}=0.5\exp\left(-\frac{V+57}{40}\right).$$

The reversal potential of Na^+^, K^+^, and Cl^−^ is formulated with the Nernst equations,

(7) $$E_{Na}=26.64\;ln\left(\frac{{\left[Na^{+}\right]}_{o}}{{\left[Na^{+}\right]}_{i}}\right),\;E_{K}=26.64\;ln\left(\frac{{\left[K^{+}\right]}_{o}}{{\left[K^{+}\right]}_{i}}\right),\;E_{Cl}=26.64\;ln\left(\frac{{\left[{Cl}^{+}\right]}_{o}}{{\left[{Cl}^{+}\right]}_{i}}\right),$$

where the subscript o and i represent the ions in the extra- and intracellular spaces.

#### 2.2.4. Dynamics of Extra- and Intracellular Ion Concentrations

In our model, the concentration of ions in the extra- and intracellular spaces is dynamic. Using the laws of conservation of charge and mass, the rate equations for the number of specific ions can be related to the currents through the channels as

(8) $$\frac{dNK_{o}^{+}}{dt}=\frac{1}{\tau }\left(\gamma {\beta \;I}_{K}-2\beta \;I_{pump}-I_{diff}-I_{glia}-2I_{gliapump}+\beta \;I_{kcc2}+\beta \;I_{nkcc1}\right){\times Vol}_{o},\;\frac{dNK_{i}^{+}}{dt}=\frac{1}{\tau }\left(-\gamma \;I_{K}+2\;I_{pump}-I_{kcc2}-I_{nkcc1}\right){\times Vol}_{i},\;\frac{dNNa_{o}^{+}}{dt}=\frac{1}{\tau }\left(\gamma \beta I_{Na}+3\beta \;I_{pump}+\beta I_{nkcc1}\right)\times {Vol}_{o},\;\frac{dNNa_{i}^{+}}{dt}=\frac{1}{\tau }\left(-\gamma I_{Na}-3I_{pump}-I_{nkcc1}\right){\times Vol}_{i},\;\frac{dNCl_{o}^{-}}{dt}=\frac{1}{\tau }\left(-\gamma \beta I_{Cl,L}+\beta I_{kcc2}+2\beta I_{nkcc1}\right){\times Vol}_{o},\;\frac{dNCl_{i}^{-}}{dt}=\frac{1}{\tau }\left(\gamma I_{Cl,L}-I_{kcc2}-2I_{nkcc1}\right){\times Vol}_{i}.$$

In these expressions, $I_{pump}$ and $I_{gliapump}$ describe the $Na^{+}/K^{+}$—ATPase flux in the neuron and astrocyte. $I_{diff}$ accounts for lateral $K^{+}$ diffusion to or from the bath solution (or blood vessel in vivo) and $I_{glia}$ represents $K^{+}$ uptake by surrounding glia through different K^+^ channels. τ converts time from seconds to milliseconds and $\beta =\frac{{Vol}_{i}}{{Vol}_{o}}$ accounts for the ratio between intracellular $\left({Vol}_{i}\right)$ and extracellular (${Vol}_{o}),$ and $\gamma =\frac{S}{{Vol}_{i}F}$ converts current in $\mu A/cm^{2}\;to\;flux\;in\;mM/s$, with S being the surface area of the cell and F Faraday’s constant.

The different fluxes incorporated in the model are functions of the intra- and/or extracellular ion concentrations and are given as

(9) $$I_{pump}=\left(\frac{\rho }{1+\exp\left(\frac{25-{\left[Na^{+}\right]}_{i}}{3}\right)}\right)\left(\frac{1}{1+\exp\left(3.5-{\left[K^{+}\right]}_{o}\right)}\right),\;I_{gliapump}=\frac{1}{3}\left(\frac{\rho }{1+\exp\left(\frac{25-{\left[Na^{+}\right]}_{gi}}{3}\right)}\right)\left(\frac{1}{1+\exp\left(3.5-{\left[K^{+}\right]}_{o}\right)}\right),\;I_{diff}=\epsilon_{k}\left({\left[K^{+}\right]}_{o}-{\left[K^{+}\right]}_{bath}\right),\;I_{glia}=\frac{G_{glia}}{1+\exp\left(\frac{18-{\left[K^{+}\right]}_{o}}{2.5}\right)},$$

where

(10) $$\rho =\frac{\rho_{max}}{1+\exp\left(\frac{20-\left[O_{2}\right]}{3}\right)}$$

represents the ${Na}^{+}/K^{+}$ pump rate, $\rho_{max}$ being the maximum Na/K pump rate, and $\left[O_{2}\right]$ is the oxygen concentration in the tissue.

(11) $$G_{glia}=\frac{G_{glia,max}}{1+\exp\left(-\frac{{\left[O_{2}\right]}_{bath}-2.5}{0.2}\right)}$$

is the rate of K^+^ uptake by glia, $G_{glia,max}$ is the maximum glial K^+^ buffering strength, and ${\left[O_{2}\right]}_{bath}$ is the *O_2_* concentration in the bath solution. These and other parameters used in the neuronal model are listed in Table 2.

(12) $$\epsilon_{k}=\frac{\epsilon_{k,max}}{1+\exp\left(-\frac{{\left[O_{2}\right]}_{bath}-2.5}{0.2}\right)}$$

is the diffusion constant of K^+^ between the bath solution and ECS, with $\epsilon_{k,max}$ being the maximal K^+^ diffusion constant.

The available O_2_ concentration in the tissue depends on the consumption by $I_{gliapump}$ and $I_{pump}$ and exchange with the bath solution. That is,

(13) $$\frac{d{\left[O_{2}\right]}_{o}}{dt}=-\alpha \left(I_{pump}+I_{gliapump}\right)+\epsilon_{o}\left({\left[O_{2}\right]}_{bath}-{\left[O_{2}\right]}_{o}\right),$$

where α is a conversion factor (mM/s→mg/sL) and $\epsilon_{0}$ is the diffusion rate for O_2_.

$I_{kcc2}$ ($K^{+}/Cl^{-}$ co-transporter) and $I_{nkcc1}(Na^{+}/K^{+}/Cl^{-}$ co-transporter) are electroneutral fluxes that regulate $\left[Cl^{-}\right]$ and are directly related to the volume regulation. These fluxes are formulated as

(14) $$I_{kcc2}=U_{kcc2}\;ln\left(\frac{{\left[K^{+}\right]}_{i}{\left[Cl^{-}\right]}_{i}}{{\left[K^{+}\right]}_{o}{\left[Cl^{-}\right]}_{o}}\right),$$

(15) $$I_{nkcc1}=U_{nkcc1}f\left({\left[K^{+}\right]}_{o}\right)ln\left(\frac{{\left[K^{+}\right]}_{i}{\left[Cl^{-}\right]}_{i}}{{\left[K^{+}\right]}_{o}{\left[Cl^{-}\right]}_{o}}\right)+ln\left(\frac{{\left[{Na}^{+}\right]}_{i}{\left[Cl^{-}\right]}_{i}}{{\left[{Na}^{+}\right]}_{o}{\left[Cl^{-}\right]}_{o}}\right),$$

where

(16) $$f\left({\left[K^{+}\right]}_{o}\right)=\frac{1}{1+\exp\left(16-{\left[K^{+}\right]}_{o}\right)}$$

regulates the channel activity and $U_{nkcc1}$ and $U_{kcc2}$ represent the maximum flux through the co-transporters.

#### 2.2.5. Volume Regulation

The effective volume of the cell ${Vol}_{i}^{*}$ is a function of the osmotic equilibrium between the intracellular and extracellular media,

(17) $${Vol}_{i}^{*}={Vol}_{i}^{0}\left(1.1029-0.1029\exp\left(\frac{\pi_{o}-\pi_{i}}{20}\right)\right),$$

where

(18) $$\pi_{o}={\left[Na^{+}\right]}_{o}+{\left[K^{+}\right]}_{o}+{\left[Cl^{-}\right]}_{o}+{\left[A^{-}\right]}_{o},$$

and

(19) $$\pi_{i}={\left[Na^{+}\right]}_{i}+{\left[K^{+}\right]}_{i}+{\left[Cl^{-}\right]}_{i}+{\left[A^{-}\right]}_{i}$$

is the sum of extra- and intracellular ions. $A^{-}$ accounts for the remaining impermeant negatively charged ions, such as proteins and phosphates. ${\left[A^{-}\right]}_{o}$ and ${\left[A^{-}\right]}_{i},$ remain constant and were calculated by assuming that the initial osmotic pressure gradient is zero.

Due to changes in the osmotic gradient, the neuronal volume becomes a dynamic variable and can be represented by a first-order differential equation,

(20) $$\frac{d{Vol}_{i}}{dt}=\frac{{Vol}_{i}^{*}-{Vol}_{i}}{250}.$$

As the neuron swells or shrinks, the extracellular volume (${Vol}_{0})$ changes accordingly,

(21) $${Vol}_{0}=\left(1+\frac{1}{\beta }\right){Vol}_{i0}-{Vol}_{i},$$

where ${Vol}_{i0}$ is the initial intracellular volume. With volume dynamics included in the model, diffusion of $K^{+}$ in the ECS is constrained by the volume fraction β. That is,

(22) $$\epsilon_{k}=\left(\frac{1}{1+\exp\left(\frac{-20+\beta }{2}\right)}\right)\left(\frac{\epsilon_{k,max}}{1+\exp\left(-\frac{{\left[O_{2}\right]}_{bath}-2.5}{0.2}\right)}\right).$$

The resulting system of equations was solved numerically in Fortran using the RK4 method. Fits to experimental data, and plotting was performed in Matlab (version R2022b, MathWorks, USA). Codes reproducing key results in this paper, including Fortran code for the full neuron/aggregation model and MATLAB script for fitting Equation (2) to the experimentally estimated values of k_2_ are available as Supplementary Information Text with this manuscript.

## 3. Results

### 3.1. Experimental Results: ThT Kinetics of Fibril vs. Oligomer Formation by Aβ42

We have previously shown that the aggregation of Aβ40, Aβ42, an Aβ40 dimer construct, and lysozyme undergoes a transition from strictly sigmoidal to progressively biphasic ThT kinetics [7,8,9,16]. The transition to biphasic ThT kinetics is directly correlated with the onset of off-pathway oligomer formation [7,8]. Here we measured the ThT kinetics of monomeric Aβ42 aggregation at five different concentrations of Aβ42 (2, 5, 10, 15, and 30 μM), and under near-physiological conditions (pH 7.4, 150 mM NaCl).

The transition from sigmoidal to biphasic kinetics is demonstrated by the sample ThT traces obtained at initial monomer concentrations of 2 μM, 5 μM, and 10 μM (Figure 2A–C). ThT traces obtained at 15 μM and 30 μM initial monomer concentrations with model fits are shown in Supplementary Information Figure S1. Given that ThT fluorescence predominantly arises from binding to ordered fibrillar β-sheet structures, we approximate the signal as proportional to fibrillar mass, while treating the oligomer-related signal significantly weaker (1% of that due to fibrils). This assumption is in line with our previous work [9], and results in close fits to the experiments performed at different monomer concentrations.

The biphasic ThT profile we observe is distinct from biphasic kinetics reported in other contexts, such as lipid-induced aggregation [73], and arises from a specific physical mechanism: concentration-dependent competition between on-pathway fibrillization and off-pathway oligomer formation [7,74]. At low monomer concentrations, monomers are consumed predominantly by on-pathway fibril nucleation and growth, producing the classical sigmoidal ThT profile. As monomer concentration increases above a critical threshold, off-pathway oligomer formation becomes kinetically competitive, diverting monomers away from the fibril-forming pathway. This produces the characteristic biphasic ThT signal—an early rise reflecting initial fibril nucleation, followed by a plateau or secondary feature as monomer depletion by oligomers slows fibril growth. Critically, this mechanism predicts that the transition from sigmoidal to biphasic should occur at a well-defined monomer concentration, which is precisely what we observe across our five-concentration series. In addition, we have shown that the increase in the initial amplitude of biphasic kinetics directly correlates with an increase in immunoreactivity of the oligomer-selective antibody A11 [75].

We exclude thermal temperature drifts in ThT fluorescence as a potential explanation for biphasic kinetics. First, all experiments were performed in a sealed, temperature-controlled fluorescence plate reader (FLUOstar Omega, BMG Labtech, Ortenberg, Germany) pre-equilibrated at 27 ± 0.3 °C for at least 30 min before plating. Second, ThT fluorescence decreases with increasing temperature. Under our conditions, this drop occurs during the first measurement cycle, as documented by our ThT-only control wells. Third, and most decisively, the biphasic character is strictly concentration-dependent: it is absent at 2 μM and becomes progressively more prominent at 5 and 10 μM (Figure 2A–C). A temperature artifact would be expected to affect all wells equally, regardless of protein concentration, whereas the concentration dependence we observe is the signature prediction of the competing-pathway mechanism.

### 3.2. Modeling the Aggregation Kinetics of Aβ42

To replicate the kinetics of Aβ42 aggregation, we adopted our previous model developed for the aggregation of an Aβ40 dimer construct (dimAβ) and hen egg white lysozyme (HewL) [7,9]. However, the aggregation kinetics of dimAβ and HewL are significantly different than the kinetics of monomeric Aβ42 (dimAβ is faster and HewL is slower than monomeric Aβ42). Thus, we allowed some of the parameters to vary to fit the model to our new data. Sample fits to the observed ThT signal are shown in Figure 2A–C, where the black, red, green, and blue lines represent the ThT signal and the concentration of off-pathway oligomers, on-pathway RFs, and the overall aggregated species, respectively.

This model can reproduce the observed transition from sigmoidal to biphasic growth as we increase initial monomer concentration. That is, the model predicts an increasingly higher probability for off-pathway oligomer formation as we increase the monomer concentration (Figure 2A–C; see also Supplementary Information Figure S1). However, as we vary the monomer concentration over a wider range, the model results in too rapid an increase in the initial fibril and/or oligomer concentration compared to the observed time traces. As reasoned in our previous work [7,9] and confirmed by experiments [16], the increasing concentration of oligomers decreases the probability of secondary nucleation due to the oligomers decorating the lateral fibril surface. In line with this reasoning, our model predicts that the secondary nucleation rate k_2_ decreases sharply with increasing monomer concentration (Figure 2D). This is consistent with the observation that at higher monomer concentration, off-pathway oligomers form more rapidly and decorate the lateral surfaces of existing fibrils, thereby inhibiting secondary nucleation [7,16].

### 3.3. Linking Neuronal Ion Homeostasis and Swelling to the Aggregation Kinetics of Aβ42

**Figure 3:**
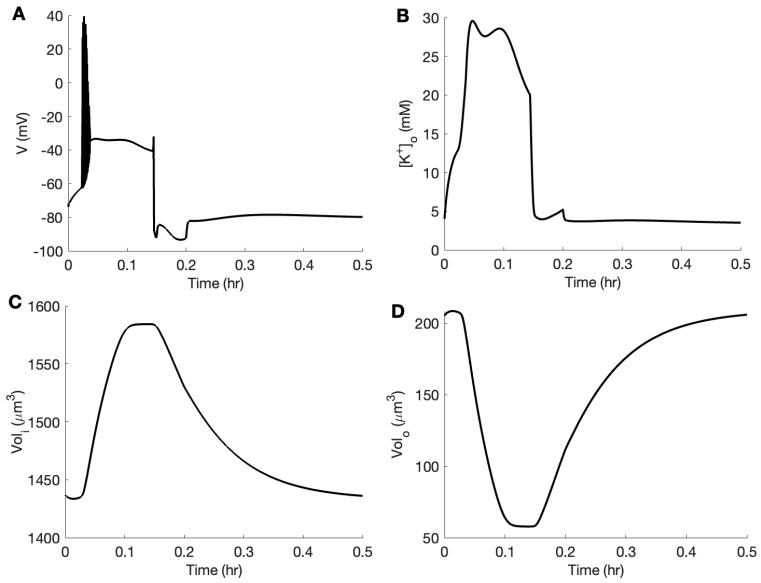
Changes in neuronal variables during SD. The membrane potential of the neuron is locked into a depolarized state during an SD (**A**), causing [K^+^]_o_ to rise (**B**). The significant changes in K^+^, Na^+^, and Cl^−^ in the intra- and extracellular spaces result in the swelling of the neuron (**C**), which causes the ECS to shrink (**D**).

Next, we determine how neuronal swelling, and consequently shrinking of the EC, during metabolic stress events such as SD affects Aβ42 aggregation kinetics. In the neuronal model, we simulate a single SD event by raising K^+^ concentration in the bath solution from 3.5 mM to 26 mM for 12 min. A sample single-cell depolarization event is shown in Figure 3A, where we raise the K^+^ concentration in the bath solution from 3.5 mM to 26 mM for the initial 12 min (0.2 h) of the simulation, followed by a restoration to a physiological value of 3.5 mM for the rest of the simulation. Note that the neuron recovers from the depolarization before we restore K^+^ to the physiological value due to the strong activation of Na^+^/K^+^-ATPase when [K^+^]_o_ (Figure 3B) and [Na^+^]_i_ rise as Na^+^/K^+^-ATPase increases like a sigmoidal function of both [K^+^]_o_ and [Na^+^]_i_ [76,77]. The shuffling of ions across the plasma membrane leads to the swelling of the neuron (Figure 3C), which consequently causes the ECS to shrink by almost 75% (Figure 3D)—in line with experimental observations [23].

**Figure 4:**
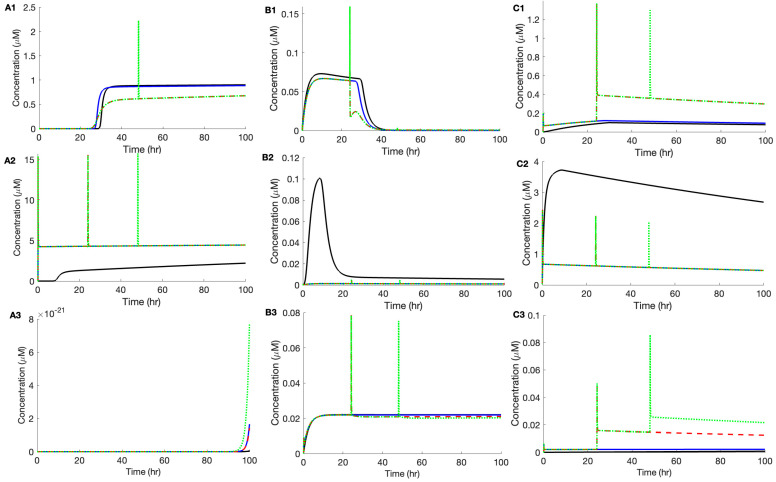
Changes in the aggregation kinetics of Aβ42 due to SD events. The time-evolution of fibril formation (**A**), off-pathway intermediate species (dimers) (**B**), and oligomers (**C**) with no (black), one (blue), two (red), and three (green) SD events, beginning at 0 h, 24 h, and 48 h, respectively, and each event lasting for 12 min. An initial monomer concentration of 1 μM (**A1**–**C1**), 5 μM (**A2**–**C2**), and 0.5 μM (**A3**–**C3**) was used.

As discussed earlier, the aggregation kinetics of Aβ42 strongly depend on initial monomer concentration (and most likely on other small aggregates such as dimers and trimers) (Figure 2). Accordingly, we hypothesize that changes in the extracellular volume (and consequently changes in the concentration of Aβ42 monomers and other aggregates) due to metabolic stress will affect the kinetics of Aβ42. To test this hypothesis, we link the model for the aggregation kinetics of Aβ42 with the neuronal model. Specifically, the concentration of Aβ42 monomers and other aggregate species changes as the extracellular volume changes. We expose the neuron to one, two, or three metabolic stress events, beginning at 0 h, 24 h, and 48 h, respectively, and each lasting for 12 min. In Figure 4A1–C1, we show results from these simulations using an initial Aβ42 monomer concentration of 1 μM. The first metabolic stress event reduces the onset time of fibril formation, decreases the concentration of intermediate species (dimers) along the off-pathway, but increases the concentration of oligomers (blue lines in Figure 4A1–C1) as compared to the no-metabolic stress scenario (black lines in Figure 4A1–C1). The second metabolic stress event mildly decreases the onset time of fibril formation with a significant reduction in its final concentration (Figure 4A1, red line). While the second event does not affect the peak concentration of the off-pathway intermediate species, it significantly decreases the duration of its rise (Figure 4B1, red line). The loss in the concentration of fibrils and intermediate species mainly results from the rise in the concentration of oligomers (Figure 4C1, red line). The third metabolic stress event, which occurs after the fibril formation has saturated, leads to a temporary rise in the concentration of fibrils, off-pathway dimers, and oligomers, but all species return to their free-metabolic stress state as soon as the event is over (green lines in Figure 4A1–C1).

We noticed that once the fibril formation plateaus, the SD event does not have any effect on the kinetics of Aβ42 aggregation (Figure 4A1–C1, green lines). To test this further, we repeated the above simulations for an initial monomer concentration of 5 μM and made the following four observations (Figure 4A2–C2). First, the effect on the fibril formation is significantly more pronounced, which rises and plateaus immediately when the first metabolic stress is applied (Figure 4A2, blue line). Second, the instantaneous concentration of off-pathway dimers is significantly lower as compared to the concentration in the absence of metabolic stress, mainly because the monomers end up in the fibrils very rapidly (Figure 4B2). Third, the concentration of oligomers decreases due to the rapid formation of fibrils (Figure 4C2). Finally, consistent with the above observation, metabolic stress does not affect the aggregation kinetics if applied after the fibril formation saturates.

As discussed above, each SD event increases the concentration of oligomers if the SD event occurs before fibril formation saturates (Figure 4C1). Accordingly, we test whether recurring metabolic stress events would raise the concentration of Aβ42 oligomers if there are negligible fibrils in the ECS. We repeat the simulation in Figure 4A1–C1 using an initial monomer concentration of 0.5 μM, where negligible fibrils are observed in the 100 h of simulation (Figure 4A3). With each consecutive metabolic stress, the concentration of Aβ42 oligomers jumps to a higher value (Figure 4C3). The increase in the oligomer concentration comes at the expense of fibril and off-pathway dimer concentrations (Figure 4B3).

Taken together, these results show that SD has a strong effect on the aggregation kinetics of Aβ42 when applied before the fibril formation plateaus. In the absence of saturating fibril concentration and low initial monomer concentration, each SD event increases the concentration of oligomers. However, at relatively high initial monomer concentration, the first SD kick starts the formation of fibrils, pushing it to saturating levels rapidly. This comes at the expense of off-pathway dimers and oligomers.

### 3.4. The Effect of Intensity and Duration of Metabolic Stress on the Aggregation Kinetics of Aβ42

**Figure 5:**
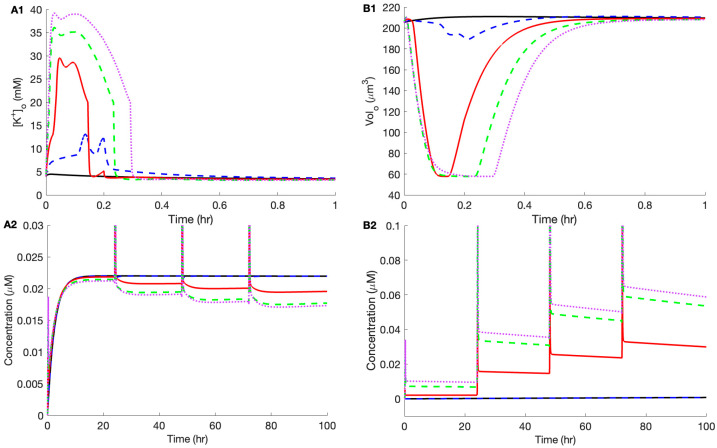
Increasing the intensity and duration of metabolic stress increases the likelihood of off-pathway aggregation. Changes in [K^+^]_o_ (**A1**) and extracellular volume (**B1**) as we raise K^+^ in the bath solution from 3.5 mM (black) to 12 mM (blue), 26 mM (red), 50 mM (green), and 60 mM (purple) for 12 min (0 to 0.2 h in the figure). Note that at high K^+^ in the bath solution, the duration for which [K^+^]_o_ stays elevated is also longer, as Na^+^/K^+^-ATPase takes longer to restore normal [K^+^]_o_. Off-pathway dimers (**A2**) and oligomers (**B2**) when the neuron is exposed to four such metabolic stress events (at 0 h, 24 h, 48 h, and 72 h), each lasting for 12 min.

Typically, an SD wave raises extracellular K^+^ nearly 20 times (to 35–60 mM) from its physiological value of 2.5–3.5 mM. Similarly, extracellular K^+^ rises during ictal epileptiform events to 10–12 mM [23]. Accordingly, we vary K^+^ in the bath from 12 mM to 60 mM to determine how the aggregation kinetics of Aβ42 change as the intensity of the metabolic stress increases. In Figure 5A1, we show one such event where we raise K^+^ in the bath for 12 min (0 h–0.2 h) to 12 mM, 26 mM, 50 mM, or 60 mM from the physiological value of 3.5 mM. Raising K^+^ in the bath increases the peak value of [K^+^]_o_ as Na^+^/K^+^-ATPase fails to keep up with the high K^+^ level (Figure 5A1). The rise in [K^+^]_o_ and other ions causes the neuron to swell, leading to a shrinkage of the ECS (Figure 5B1). The duration for which [K^+^]_o_ stays elevated, and the ECS remains shrunk, also increases progressively with increasing K^+^ in the bath as Na^+^/K^+^-ATPase takes longer to restore the K^+^ and Na^+^ gradients.

Next, we apply four such metabolic stress events (at 0 h, 24 h, 48 h, and 72 h) of different intensities by raising K^+^ in the bath for 12 min from its physiological value (Figure 5A1,B1). Exposing the neuron to four seizure-like events (raising K^+^ in the bath to 12 mM during each event) does not have any significant effect on the aggregation kinetics, as the time traces of both off-pathway dimers (Figure 5A2) and oligomers (Figure 5B2) remain unchanged. This is clear from the blue lines (seizure-like events) lying on top of the black lines (control with fixed bath K^+^ of 3.5 mM throughout the simulation). We emphasize that the ~12 mM extracellular K^+^ used in this simulation is typical of seizure-like events and is much lower than that associated with SD events [71].

As discussed above, increasing bath K^+^ from 3.5 mM to 26 mM during each event (typical of [K^+^]_o_ levels during SD associated with migraine aura, where the cortex is well perfused) significantly affects the aggregation kinetics, where the level of oligomers consistently rises with each stress event (red line in Figure 5B2). The rise in oligomer concentration is linked with a drop in the off-pathway dimers (Figure 5A2). The concentration of oligomers increases further as we increase bath K^+^ from 3.5 mM to 50 mM or 60 mM during each event, leading to [K^+^]_o_ values that are typically associated with SD during stroke, ischemia, and traumatic brain injury, where the tissue energy is compromised.

### 3.5. Pre-Existing Nuclei or Off-Pathway Dimers Significantly Accelerate the Aggregation Kinetics of Aβ42

**Figure 6:**
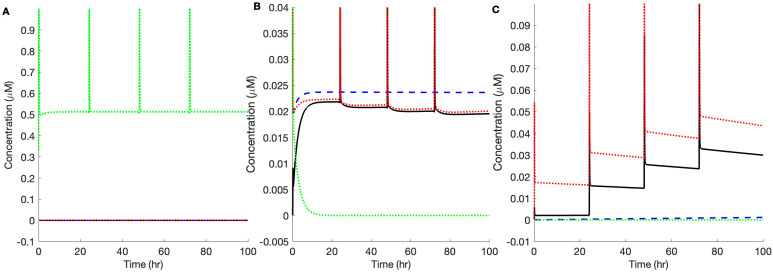
Pre-existing off-pathway dimers exacerbate the formation of oligomers due to metabolic stress, whereas pre-existing on-pathway nuclei promote the formation of fibrils, suppressing the formation of off-pathway species. Changes in fibril (**A**), off-pathway dimers (**B**), and oligomer (**C**) formation when the neuron is exposed to four SD events without pre-existing dimers or nuclei (black lines), with 0.01 μM pre-existing off-pathway dimers (red), with pre-existing 0.01 μM off-pathway dimers and 0.001 μM on-pathway nuclei (green), and with 0.01 μM pre-existing off-pathway dimers but no metabolic stress (blue). An initial monomer concentration of 0.5 μM is used, and the four metabolic stress events are applied by raising K^+^ in the bath solution from 3.5 mM to 26 mM for 12 min at 0 h, 24 h, 48 h, and 72 h.

Another question we sought to address was how pre-existing aggregates in the ECS influenced aggregation kinetics during SD. Specifically, we examined the effect of pre-existing on-pathway nuclei or off-pathway dimers. In these simulations, we first initialized the system with 0.01 μM off-pathway dimers in addition to 0.5 μM monomers. Introducing pre-existing off-pathway dimers without applying metabolic stress increased the dimer concentration but did not promote the formation of fibrils or oligomers (blue lines in Figure 6A–C). Next, we repeated the same simulation while exposing the neuron to four metabolic stress events at 0, 24, 48, and 72 h. Under this condition, the number of fibrils remained essentially unchanged, whereas the number of oligomers increased substantially compared with the scenario in which four metabolic stress events were applied (at 0, 24, 48, and 72 h) without pre-existing off-pathway dimers (red vs. black lines in Figure 6A, 6C, respectively). In contrast, the concentration of off-pathway dimers was initially higher due to the presence of the pre-existing dimers, but over time it converged to levels comparable to those observed in the absence of pre-existing dimers (Figure 6B, red vs. black line).

Next, we introduced pre-existing on-pathway nuclei (0.001 μM) in addition to the off-pathway dimers (0.01 μM). Under this protocol, fibril formation was markedly accelerated and reached a plateau immediately following the first SD event. The presence of pre-existing nuclei effectively depleted the off-pathway dimers and suppressed oligomer formation (green lines in Figure 6A–C). This is due to the rapid rate of fibril elongation past the nucleation stage. Finally, we simulated the same initial conditions (0.001 μM on-pathway nuclei and 0.01 μM off-pathway dimers) in the absence of metabolic stress. Even without metabolic stress, fibril concentration rapidly reached saturation. These results suggest that the presence of pre-existing nuclei alone is sufficient to drive fibril formation to saturation, leaving little or no room for metabolic stress to further exacerbate aggregation.

## 4. Discussion

In this study, we present a multiscale framework that links neuronal ion dynamics, volume regulation, and amyloid aggregation to investigate how metabolic stress influences the kinetics of Aβ42 aggregation in the ECS. Our results provide mechanistic insight into how SD and related pathological events can create transient but significant shifts in the extracellular microenvironment that alter aggregation kinetics. A central finding of this work is that neuronal swelling during SD leads to substantial shrinkage of the ECS, resulting in a rapid increase in the concentration of Aβ42. This rise in the concentration alone is sufficient to shift aggregation kinetics, promoting earlier onset of aggregation and altering the balance between on-pathway fibril formation and off-pathway oligomerization. Importantly, our results show that the timing of metabolic stress relative to the aggregation trajectory is critical. When SD occurs before fibril formation saturates, it can significantly accelerate aggregation and bias the system toward either oligomer formation or fibrillization, depending on the initial monomer concentration. In contrast, once fibril formation has reached a plateau, metabolic stress has minimal impact on aggregation kinetics, suggesting that the system becomes kinetically trapped in a stable state. This stable state is a direct result of a key assumption in the model, namely that fibrils are stable and irreversible species based on fits to the experimental data.

At low initial monomer concentrations, repeated metabolic stress events lead to a progressive accumulation of off-pathway oligomers. This is particularly significant given the widely recognized neurotoxicity of soluble oligomeric Aβ species. In contrast, at higher monomer concentrations, metabolic stress rapidly drives fibril formation, effectively depleting monomers and suppressing oligomer formation. These findings highlight a nonlinear and concentration-dependent response of the aggregation kinetics to metabolic stress, suggesting that small differences in baseline Aβ42 levels could lead to markedly different pathological outcomes under similar stress conditions.

Another key result from this study is the role of pre-existing small aggregate species in modulating the system’s response to metabolic stress. The presence of off-pathway dimers amplifies oligomer formation during SD, whereas the presence of on-pathway nuclei promotes rapid fibrillization and suppresses oligomer accumulation. These findings emphasize the importance of initial conditions and suggest that even small amounts of pre-existing aggregates can strongly influence disease progression. In particular, the presence of fibril seeds appears to buffer the system against further perturbations, while small oligomeric seeds exacerbate pathological aggregation under stress. This result is of particular importance in situations where clusters of SD events occur, as is usually observed in TBI and recurrent migraines [28,32,38]. Under such a scenario, the first one or two SD events might promote the formation of smaller species, which then accelerate the formation of oligomers or fibrils by the later SD events. This is something that warrants further investigation in the future.

The intensity and duration of metabolic stress also play a crucial role in promoting aggregation. Increasing extracellular K^+^ levels—mimicking conditions observed in severe SD associated with stroke or TBI—not only enhances ECS shrinkage but also prolongs its duration. This leads to sustained increases in Aβ42 concentration and a corresponding increase in oligomer formation. In contrast, milder stress conditions, such as those associated with seizure-like activity, have negligible effects on aggregation kinetics. This reveals a threshold-like relationship between stress intensity and aggregation outcome. At 12 mM K^+^ in the bath solution (seizure-like events), the Na^+^/K^+^-ATPase recovers rapidly, producing only brief and modest ECS shrinkage that is insufficient to meaningfully elevate Aβ42 concentration. This is reflected in the blue curves in Figure 5A2 and 5B2 being indistinguishable from the no-stress control—suggesting that the sporadic ictal events associated with epilepsy may not, in isolation, be sufficient to drive pathological aggregation via this mechanism.

In contrast, SD-level K^+^ elevation (bath K^+^ ≥ 26 mM) crosses a threshold at which Na^+^/K^+^-ATPase recovery is significantly slowed, producing prolonged ECS shrinkage and sustaining elevated Aβ42 concentration for minutes. This threshold behavior is a direct consequence of the sigmoidal dependence of Na^+^/K^+^-ATPase activity on [K^+^]_o_ and [Na^+^]_i_. At SD-level K^+^, the pump rate is insufficient to restore gradients in real time, generating an extended aggregation-permissive window. At bath K^+^ = 50–60 mM (stroke/TBI conditions), this window is further extended, producing not only a higher peak Aβ42 concentration but a longer duration of sustained elevation. This temporal prolongation may be particularly important for fibril nucleation, which requires sustained supersaturation. These findings have direct clinical relevance: in TBI and severe migraine with aura, where SD clusters persist for hours or days [35], the cumulative duration of aggregation-permissive conditions may be sufficient to produce significant and irreversible shifts in the Aβ42 aggregation landscape.

While the model captures key aspects of Aβ42 aggregation under dynamic extracellular conditions, it represents a deliberate simplification designed to isolate the contribution of ECS volume changes—a first-principles mechanism directly resulting from neuronal swelling during SD. Several important mechanisms are not included in the current framework, and we expect all of them to act cooperatively with the volume effect, meaning our predictions represent conservative lower bounds on SD-induced aggregation enhancement.

First, SD causes pH in the ECS to drop from ~7.4 to as low as 6.1 near the ischemic core [23,68]. Independent experimental work demonstrates that mildly acidic conditions (pH 6.0–6.5) markedly promote Aβ42 oligomerization and shift the aggregation equilibrium toward off-pathway species [18,20,21]. Including pH effects would amplify our predicted oligomer accumulation, potentially several-fold.

Second, extracellular K^+^ rises from ~3.5 mM to 35–60 mM during SD, and Na^+^ and Cl^−^ also undergo dramatic changes [23]. Our preliminary experiments show that while increasing NaCl does not affect oligomer or fibril formation of Aβ42 in a biphasic regime, it decreases the lag time for fibril formation in the pure sigmoidal regime. Similarly, we observed that KCl does not directly affect oligomer formation, but it seems to promote fibril formation by reducing the lag time of fibril formation. An independent study also showed that altered ionic strength affects the aggregation kinetics of Aβ40 in a concentration- and pH-dependent manner [19]. These electrostatic effects are expected to cooperate with the volume-driven concentration increase.

Third, the current framework does not explicitly include diffusion of Aβ42 species within the ECS. The tortuosity of the ECS and diffusion constraints are themselves altered during swelling, which could further modulate aggregation kinetics [78]. Incorporating spatial diffusion and heterogeneous ECS structure would provide a more complete description of aggregation dynamics.

Fourth, the model does not include Aβ42 production or glymphatic clearance. Hypoxia and ischemia increase BACE1 expression 2–3 fold, elevating Aβ42 production rates beyond our model’s scope [29,31,38]. Concurrently, astrocytic swelling—which persists for hours after an SD wave [79]—impairs Aβ42 clearance via astrocytic LRP1 and the glymphatic pathway [80,81], compounding ECS concentration increases. Furthermore, the ECS diffusion tortuosity increases during swelling [78], which would further reduce local clearance rates.

Finally, the aggregation model uses a mean-field, spatially homogeneous representation. Kinetic Monte Carlo simulations could better capture stochastic nucleation events and spatial heterogeneity at the microscale. These processes are expected to interact with the concentration-driven effects described here and are the subject of our future work.

Despite these limitations, our findings provide a unifying framework for understanding how acute metabolic stress events may act as triggers for pathological Aβ aggregation. By linking well-characterized physiological processes—such as ion dysregulation, neuronal swelling, and ECS shrinkage—to aggregation kinetics derived from extensive experimental data, this work bridges a critical gap between cellular physiology and molecular pathology. Importantly, the results suggest that recurrent SD events, as observed in TBI, stroke, and migraine, could serve as repeated perturbations that progressively drive the system toward pathological states. Overall, this study supports the hypothesis that metabolic stress and SD are not merely correlates of neurodegenerative disease but may play a direct mechanistic role in initiating and modulating Aβ aggregation.

## 5. Conclusions

In this work, we developed a multiscale computational framework integrating neuronal ion homeostasis, extracellular volume dynamics, and Aβ42 aggregation kinetics to investigate how metabolic stress—specifically SD—modulates the formation of amyloid aggregates in the brain’s ECS. Our central finding is that neuronal swelling during SD causes substantial ECS shrinkage, which alone is sufficient to significantly accelerate Aβ42 aggregation and shift the balance between on-pathway fibril formation and off-pathway oligomerization. The direction and magnitude of this shift depend critically on: (1) the initial monomer concentration, (2) the timing of metabolic stress relative to aggregation progression, (3) the presence and type of pre-existing aggregate species, and (4) the intensity and duration of the stress event.

Specifically, (1) at low monomer concentration, recurring SD events progressively accumulate neurotoxic off-pathway oligomers; (2) at high monomer concentration, a single SD event can drive rapid fibrillization to saturation; (3) pre-existing fibril nuclei suppress oligomer formation, while pre-existing dimers amplify it; and (4) only SD-level (not seizure-level) metabolic disturbances cross the threshold required to significantly impact aggregation. These findings provide a mechanistic basis for the epidemiological link between conditions involving SD—TBI, stroke, hypoxia, migraine—and elevated AD risk, and establish a quantitative framework for future investigation of SD as a trigger for early-stage Aβ42 pathology.

## Figures and Tables

**Table 1:** Fitted kinetic rate constants for Aβ42 aggregation model.

Parameter	Meaning	Value	Source
$a_{1}$	Primary nucleation rate	$1.98\times 10^{-41}\;M^{-4}{hr}^{-1}$	This work
$b_{1}$	Dissociation rate of primary nuclei	$3.96{\times 10}^{-4}\;{hr}^{-1}$	[9]
a	RF formation rate	$1.98\times 10^{11}\;M^{-1}{hr}^{-1}$	[9]
b	RF dissociation rate	$1.98{\times 10}^{7}\;{hr}^{-1}$	[9]
$\alpha_{1}$	Dimer formation rate	$1.8\times 10^{4}\;M^{-1}{hr}^{-1}$	This work
$\beta_{1}$	Dimer dissociation rate	$3.6\times 10^{-1}\;{hr}^{-1}$	This work
α	gOs formation rate	$3.6\times 10^{46}\;M^{-8}{hr}^{-1}$	This work
β	gOs dissociation rate	${3.6\times 10}^{-3}\;{hr}^{-1}$	This work

**Table 2:** Parameters used in the neuron model.

Parameter	Meaning	Value	Source
C	Membrane capacitance	$1\;\mu F/cm^{2}$	[71]
$G_{Na}$	Maximal conductance of sodium current	$30\;mS/cm^{2}$	[71]
$G_{K}$	Maximal conductance of potassium current	$25\;mS/cm^{2}$	[71]
$G_{Na,L}$	Conductance of leak sodium current	$0.0247\;mS/cm^{2}$	[71]
$G_{K,L}$	Conductance of leak potassium current	$0.05\;mS/cm^{2}$	[71]
$G_{Cl,L}$	Conductance of leak chloride current	$0.1\;mS/cm^{2}$	[71]
$\beta_{0}$	Ratio of the initial intra-/extracellular volume	7	[71]
$G_{glia,max}$	Maximal glial uptake strength of potassium	5 mM/s	[71]
$\epsilon_{k,max}$	Maximal potassium diffusion rate	$0.25\;s^{-1}$	[71]
${\left[K^{+}\right]}_{bath}$	Normal bath potassium concentration	3.5 mM	[71]
$\epsilon_{0}$	Oxygen diffusion rate	$0.17\;s^{-1}$	[71]
α	Conversion factor	5.3 g/mol	[71]
$U_{kcc2}$	Maximal KCC2 cotransporter strength	0.3 mM/s	[71]
$U_{nkcc1}$	Maximal NKCC1 cotransporter strength	0.1 mM/s	[71]
${\left[O_{2}\right]}_{bath}$	Normal bath oxygen concentration	32 mg/L	[71]
$\rho_{max}$	Maximal Na/K pump rate	0.8 mM/s	[71]
${\left[Na^{+}\right]}_{gi}$	Sodium concentration in glia	18 mM	[71]
${\left[A^{-}\right]}_{i}$	Intracellular impermeable anions	132 mM	[71]
${\left[A^{-}\right]}_{o}$	Extracellular impermeable anions	18 mM	[71]

## Data Availability

The original contributions presented in this study are included in the article/Supplementary Materials. Further inquiries can be directed to the corresponding author.

## Supplementary Materials

The following supporting information can be downloaded at: https://www.mdpi.com/article/10.3390/biom16071053/s1, The Fortran and Matlab codes reproducing the key results in this paper, along with instructions to use the codes. Figure S1: Biphasic Aβ42 aggregation kinetics at higher monomer concentration along with model fits. Traces at 15 μM (A) and 30 μM (B), where the black, red, green, and blue lines represent experimental data, off-pathway oligomers, on-pathway RFs, and the overall aggregated species given by the model, respectively. Experiments at each monomer concentration were repeated three times, as shown by the three black lines in each panel.

## References

[1] Hardy J., Selkoe D.J. (2002). The amyloid hypothesis of Alzheimer’s disease: Progress and problems on the road to therapeutics. Science.

[2] Hardy J.A., Higgins G.A. (1992). Alzheimer’s disease: The amyloid cascade hypothesis. Science.

[3] Brisson C.D., Andrew R.D. (2012). A neuronal population in hypothalamus that dramatically resists acute ischemic injury compared to neocortex. J. Neurophysiol..

[4] Cole S.L., Vassar R. (2007). The Alzheimer’s disease β-secretase enzyme, BACE1. Mol. Neurodegener..

[5] Wolfe M.S., Xia W., Ostaszewski B.L., Diehl T.S., Kimberly W.T., Selkoe D.J. (1999). Two transmembrane aspartates in presenilin-1 required for presenilin endoproteolysis and γ-secretase activity. Nature.

[6] De Strooper B., Annaert W., Cupers P., Saftig P., Craessaerts K., Mumm J.S., Schroeter E.H., Schrijvers V., Wolfe M.S., Ray W.J. (1999). A presenilin-1-dependent γ-secretase-like protease mediates release of Notch intracellular domain. Nature.

[7] Hasecke F., Miti T., Perez C., Barton J., Schölzel D., Gremer L., Grüning C.S., Matthews G., Meisl G., Knowles T.P. (2018). Origin of metastable oligomers and their effects on amyloid fibril self-assembly. Chem. Sci..

[8] Miti T., Mulaj M., Schmit J.D., Muschol M. (2015). Stable, metastable, and kinetically trapped amyloid aggregate phases. Biomacromolecules.

[9] Perez C., Miti T., Hasecke F., Meisl G., Hoyer W., Muschol M., Ullah G. (2019). Mechanism of fibril and soluble oligomer formation in amyloid beta and hen egg white lysozyme proteins. J. Phys. Chem. B.

[10] Arriagada P.V., Growdon J.H., Hedley-Whyte E.T., Hyman B.T. (1992). Neurofibrillary tangles but not senile plaques parallel duration and severity of Alzheimer’s disease. Neurology.

[11] Demuro A., Mina E., Kayed R., Milton S.C., Parker I., Glabe C.G. (2005). Calcium dysregulation and membrane disruption as a ubiquitous neurotoxic mechanism of soluble amyloid oligomers. J. Biol. Chem..

[12] Tomic J.L., Pensalfini A., Head E., Glabe C.G. (2009). Soluble fibrillar oligomer levels are elevated in Alzheimer’s disease brain and correlate with cognitive dysfunction. Neurobiol. Dis..

[13] Benilova I., Karran E., De Strooper B. (2012). The toxic Aβ oligomer and Alzheimer’s disease: An emperor in need of clothes. Nat. Neurosci..

[14] Kayed R., Head E., Thompson J.L., McIntire T.M., Milton S.C., Cotman C.W., Glabe C.G. (2003). Common structure of soluble amyloid oligomers implies common mechanism of pathogenesis. Science.

[15] Demuro A., Smith M., Parker I. (2011). Single-channel Ca2+ imaging implicates Aβ1–42 amyloid pores in Alzheimer’s disease pathology. J. Cell Biol..

[16] Hasecke F., Niyangoda C., Borjas G., Pan J., Matthews G., Muschol M., Hoyer W. (2021). Protofibril–Fibril Interactions Inhibit Amyloid Fibril Assembly by Obstructing Secondary Nucleation. Angew. Chem..

[17] Stine W.B., Dahlgren K.N., Krafft G.A., LaDu M.J. (2003). In vitro characterization of conditions for amyloid-β peptide oligomerization and fibrillogenesis. J. Biol. Chem..

[18] Atwood C.S., Moir R.D., Huang X., Scarpa R.C., Bacarra N.M.E., Romano D.M., Hartshorn M.A., Tanzi R.E., Bush A.I. (1998). Dramatic aggregation of Alzheimer Aβ by Cu (II) is induced by conditions representing physiological acidosis. J. Biol. Chem..

[19] Campos-Ramírez A., Márquez M., Quintanar L., Rojas-Ochoa L.F. (2017). Effect of ionic strength on the aggregation kinetics of the amidated amyloid beta peptide Aβ (1-40) in aqueous solutions. Biophys. Chem..

[20] Gorman P.M., Yip C.M., Fraser P.E., Chakrabartty A. (2003). Alternate aggregation pathways of the Alzheimer β-amyloid peptide: Aβ association kinetics at endosomal pH. J. Mol. Biol..

[21] Schützmann M.P., Hasecke F., Bachmann S., Zielinski M., Hänsch S., Schröder G.F., Zempel H., Hoyer W. (2021). Endo-lysosomal Aβ concentration and pH trigger formation of Aβ oligomers that potently induce Tau missorting. Nat. Commun..

[22] Silvers R., Colvin M.T., Frederick K.K., Jacavone A.C., Lindquist S., Linse S., Griffin R.G. (2017). Aggregation and fibril structure of AβM01–42 and Aβ1–42. Biochemistry.

[23] Dreier J.P., Reiffurth C. (2015). The stroke-migraine depolarization continuum. Neuron.

[24] Thapaliya P., Pape N., Rose C.R., Ullah G. (2023). Modeling the heterogeneity of sodium and calcium homeostasis between cortical and hippocampal astrocytes and its impact on bioenergetics. Front. Cell. Neurosci..

[25] Meisl G., Kirkegaard J.B., Arosio P., Michaels T.C., Vendruscolo M., Dobson C.M., Linse S., Knowles T.P. (2016). Molecular mechanisms of protein aggregation from global fitting of kinetic models. Nat. Protoc..

[26] Cohen S.I., Linse S., Luheshi L.M., Hellstrand E., White D.A., Rajah L., Otzen D.E., Vendruscolo M., Dobson C.M., Knowles T.P. (2013). Proliferation of amyloid-β42 aggregates occurs through a secondary nucleation mechanism. Proc. Natl. Acad. Sci. USA.

[27] Ayata C., Lauritzen M. (2015). Spreading depression, spreading depolarizations, and the cerebral vasculature. Physiol. Rev..

[28] Somjen G.G. (2001). Mechanisms of spreading depression and hypoxic spreading depression-like depolarization. Physiol. Rev..

[29] Guglielmotto M., Aragno M., Autelli R., Giliberto L., Novo E., Colombatto S., Danni O., Parola M., Smith M.A., Perry G. (2009). The up-regulation of BACE1 mediated by hypoxia and ischemic injury: Role of oxidative stress and HIF1α. J. Neurochem..

[30] Gren M., Shahim P., Lautner R., Wilson D.H., Andreasson U., Norgren N., Blennow K., Zetterberg H. (2016). Blood biomarkers indicate mild neuroaxonal injury and increased amyloid β production after transient hypoxia during breath-hold diving. Brain Inj..

[31] Zhang X., Zhou K., Wang R., Cui J., Lipton S.A., Liao F.-F., Xu H., Zhang Y.-w. (2007). Hypoxia-inducible factor 1α (HIF-1α)-mediated hypoxia increases BACE1 expression and β-amyloid generation. J. Biol. Chem..

[32] Kramer D.R., Fujii T., Ohiorhenuan I., Liu C.Y. (2016). Cortical spreading depolarization: Pathophysiology, implications, and future directions. J. Clin. Neurosci..

[33] Lauritzen M., Dreier J.P., Fabricius M., Hartings J.A., Graf R., Strong A.J. (2011). Clinical relevance of cortical spreading depression in neurological disorders: Migraine, malignant stroke, subarachnoid and intracranial hemorrhage, and traumatic brain injury. J. Cereb. Blood Flow Metab..

[34] Hartings J.A., Shuttleworth C.W., Kirov S.A., Ayata C., Hinzman J.M., Foreman B., Andrew R.D., Boutelle M.G., Brennan K., Carlson A.P. (2017). The continuum of spreading depolarizations in acute cortical lesion development: Examining Leao’s legacy. J. Cereb. Blood Flow Metab..

[35] Dreier J.P. (2011). The role of spreading depression, spreading depolarization and spreading ischemia in neurological disease. Nat. Med..

[36] Pensato U., Ospel J.M., Lemale C.L., Hartings J.A., Romoli M., Sacco S., Dreier J.P. (2026). Spreading depolarization: A wave that precedes and drives cerebral ischemic cell death—Target for neuroprotection. J. Cereb. Blood Flow Metab..

[37] Díaz-Pérez A., De Eulate N.A., Masvidal-Codina E., Illa X., Navarro X., Guimerà-Brunet A., Jiménez-Altayó F., Penas C. (2026). Cortical spreading depolarizations in stroke: Mechanisms, neuroprotective interventions, and monitoring techniques. GeroScience.

[38] Blasko I., Beer R., Bigl M., Apelt J., Franz G., Rudzki D., Ransmayr G., Kampfl A., Schliebs R. (2004). Experimental traumatic brain injury in rats stimulates the expression, production and activity of Alzheimer’s disease β-secretase (BACE-1). J. Neural Transm..

[39] Pluta R., Jabłoński M., Ułamek-Kozioł M., Kocki J., Brzozowska J., Januszewski S., Furmaga-Jabłońska W., Bogucka-Kocka A., Maciejewski R., Czuczwar S.J. (2013). Sporadic Alzheimer’s disease begins as episodes of brain ischemia and ischemically dysregulated Alzheimer’s disease genes. Mol. Neurobiol..

[40] Morton R.E., St John P.D., Tyas S.L. (2019). Migraine and the risk of all-cause dementia, Alzheimer’s disease, and vascular dementia: A prospective cohort study in community-dwelling older adults. Int. J. Geriatr. Psychiatry.

[41] Kim H.-K., Park S., Kim S.-W., Jin Y., Lee H., Hong J.Y., Hong I., Baek M.S. (2025). Associations between traumatic brain injury and the prevalence of Alzheimer’s disease dementia and behavioral and psychological symptoms of dementia: A retrospective cohort study. J. Prev. Alzheimer’s Dis..

[42] Roe C.M., Fagan A.M., Grant E.A., Hassenstab J., Moulder K.L., Dreyfus D.M., Sutphen C.L., Benzinger T.L., Mintun M.A., Holtzman D.M. (2013). Amyloid imaging and CSF biomarkers in predicting cognitive impairment up to 7.5 years later. Neurology.

[43] Vos S.J., Xiong C., Visser P.J., Jasielec M.S., Hassenstab J., Grant E.A., Cairns N.J., Morris J.C., Holtzman D.M., Fagan A.M. (2013). Preclinical Alzheimer’s disease and its outcome: A longitudinal cohort study. Lancet Neurol..

[44] Tarawneh R., D’Angelo G., Macy E., Xiong C., Carter D., Cairns N.J., Fagan A.M., Head D., Mintun M.A., Ladenson J.H. (2011). Visinin-like protein-1: Diagnostic and prognostic biomarker in Alzheimer disease. Ann. Neurol..

[45] Nordström A., Nordström P. (2018). Traumatic brain injury and the risk of dementia diagnosis: A nationwide cohort study. PLoS Med..

[46] Alzheimer’s Association (2002). Traumatic Brain Injury (TBI). https://www.alz.org/alzheimers-dementia/what-is-dementia/related_conditions/traumatic-brain-injury.

[47] Nemetz P.N., Leibson C., Naessens J.M., Beard M., Kokmen E., Annegers J.F., Kurland L.T. (1999). Traumatic brain injury and time to onset of Alzheimer’s disease: A population-based study. Am. J. Epidemiol..

[48] Iacono D., Raiciulescu S., Olsen C., Perl D.P. (2021). Traumatic brain injury exposure lowers age of cognitive decline in AD and non-AD conditions. Front. Neurol..

[49] Rostowsky K.A., Irimia A. (2021). Acute cognitive impairment after traumatic brain injury predicts the occurrence of brain atrophy patterns similar to those observed in Alzheimer’s disease. GeroScience.

[50] Association A.s. (2019). 2019 Alzheimer’s disease facts and figures. Alzheimer’s Dement..

[51] Mies G., Paschen W. (1984). Regional changes of blood flow, glucose, and ATP content determined on brain sections during a single passage of spreading depression in rat brain cortex. Exp. Neurol..

[52] Hansen A.J., Zeuthen T. (1981). Extracellular ion concentrations during spreading depression and ischemia in the rat brain cortex. Acta Physiol. Scand..

[53] Andrew R.D., Farkas E., Hartings J.A., Brennan K., Herreras O., Müller M., Kirov S.A., Ayata C., Ollen-Bittle N., Reiffurth C. (2022). Questioning glutamate excitotoxicity in acute brain damage: The importance of spreading depolarization. Neurocritical Care.

[54] Andrew R.D., Hartings J.A., Ayata C., Brennan K., Dawson-Scully K.D., Farkas E., Herreras O., Kirov S., Müller M., Ollen-Bittle N. (2022). The critical role of spreading depolarizations in early brain injury: Consensus and contention. Neurocritical Care.

[55] Rungta R.L., Choi H.B., Tyson J.R., Malik A., Dissing-Olesen L., Lin P.J., Cain S.M., Cullis P.R., Snutch T.P., MacVicar B.A. (2015). The cellular mechanisms of neuronal swelling underlying cytotoxic edema. Cell.

[56] Sonn J., Mayevsky A. (2000). Effects of brain oxygenation on metabolic, hemodynamic, ionic and electrical responses to spreading depression in the rat. Brain Res..

[57] Lauritzen M. (1987). Regional cerebral blood flow during cortical spreading depression in rat brain: Increased reactive hyperperfusion in low-flow states. Acta Neurol. Scand..

[58] Piper R.D., Lambert G.A., Duckworth J.W. (1991). Cortical blood flow changes during spreading depression in cats. Am. J. Physiol.-Heart Circ. Physiol..

[59] Jing J., Aitken P.G., Somjen G.G. (1994). Interstitial volume changes during spreading depression (SD) and SD-like hypoxic depolarization in hippocampal tissue slices. J. Neurophysiol..

[60] Chesler M. (2003). Regulation and modulation of pH in the brain. Physiol. Rev..

[61] Chesler M. (2005). Failure and function of intracellular pH regulation in acute hypoxic-ischemic injury of astrocytes. Glia.

[62] Menyhárt Á., Zölei-Szénási D., Puskás T., Makra P., Orsolya M.T., Szepes B.É., Tóth R., Ivánkovits-Kiss O., Obrenovitch T.P., Bari F. (2017). Spreading depolarization remarkably exacerbates ischemia-induced tissue acidosis in the young and aged rat brain. Sci. Rep..

[63] Rogers M.L., Leong C.L., Gowers S.A., Samper I.C., Jewell S.L., Khan A., McCarthy L., Pahl C., Tolias C.M., Walsh D.C. (2017). Simultaneous monitoring of potassium, glucose and lactate during spreading depolarization in the injured human brain–Proof of principle of a novel real-time neurochemical analysis system, continuous online microdialysis. J. Cereb. Blood Flow Metab..

[64] Taylor D.L., Obrenovitch T.P., Symon L. (1996). Changes in extracellular acid-base homeostasis in cerebral ischemia. Neurochem. Res..

[65] Pérez-Pinzón M., Tao L., Nicholson C. (1995). Extracellular potassium, volume fraction, and tortuosity in rat hippocampal CA1, CA3, and cortical slices during ischemia. J. Neurophysiol..

[66] Song M., Yu S.P. (2014). Ionic regulation of cell volume changes and cell death after ischemic stroke. Transl. Stroke Res..

[67] Müller M., Somjen G.G. (2000). Na+ and K+ concentrations, extra-and intracellular voltages, and the effect of TTX in hypoxic rat hippocampal slices. J. Neurophysiol..

[68] Mutch W., Hansen A. (1984). Extracellular pH changes during spreading depression and cerebral ischemia: Mechanisms of brain pH regulation. J. Cereb. Blood Flow Metab..

[69] Jin S., Kedia N., Illes-Toth E., Haralampiev I., Prisner S., Herrmann A., Wanker E.E., Bieschke J. (2016). Amyloid-β (1–42) aggregation initiates its cellular uptake and cytotoxicity. J. Biol. Chem..

[70] Powers E.T., Powers D.L. (2008). Mechanisms of protein fibril formation: Nucleated polymerization with competing off-pathway aggregation. Biophys. J..

[71] Wei Y., Ullah G., Schiff S.J. (2014). Unification of neuronal spikes, seizures, and spreading depression. J. Neurosci..

[72] Hodgkin A.L., Huxley A.F. (1952). A quantitative description of membrane current and its application to conduction and excitation in nerve. J. Physiol..

[73] Brown J.W., Meisl G., Knowles T.P., Buell A.K., Dobson C.M., Galvagnion C. (2018). Kinetic barriers to α-synuclein protofilament formation and conversion into mature fibrils. Chem. Commun..

[74] Muschol M., Hoyer W. (2023). Amyloid oligomers as on-pathway precursors or off-pathway competitors of fibrils. Front. Mol. Biosci..

[75] Karunarathne K., Kee T.R., Jeon H., Cazzaro S., Gamage Y.I., Pan J., Woo J.-A., Kang D.E., Muschol M. (2024). Crystal violet selectively detects aβ oligomers but not fibrils in vitro and in Alzheimer’s disease brain tissue. Biomolecules.

[76] Larsen B.R., Holm R., Vilsen B., MacAulay N. (2016). Glutamate transporter activity promotes enhanced Na+/K+-ATPase-mediated extracellular K+ management during neuronal activity. J. Physiol..

[77] Larsen B.R., Assentoft M., Cotrina M.L., Hua S.Z., Nedergaard M., Kaila K., Voipio J., MacAulay N. (2014). Contributions of the Na+/K+-ATPase, NKCC1, and Kir4. 1 to hippocampal K+ clearance and volume responses. Glia.

[78] Syková E., Nicholson C. (2008). Diffusion in brain extracellular space. Physiol. Rev..

[79] Risher W.C., Croom D., Kirov S.A. (2012). Persistent astroglial swelling accompanies rapid reversible dendritic injury during stroke-induced spreading depolarizations. Glia.

[80] Liu C.-C., Hu J., Zhao N., Wang J., Wang N., Cirrito J.R., Kanekiyo T., Holtzman D.M., Bu G. (2017). Astrocytic LRP1 mediates brain Aβ clearance and impacts amyloid deposition. J. Neurosci..

[81] Ries M., Sastre M. (2016). Mechanisms of Aβ clearance and degradation by glial cells. Front. Aging Neurosci..

