# Supplementary material for: Shrinking of Extracellular Space During Metabolic Stress Accelerates Amyloid-β Aggregation
Source: Biomolecules. 2026 Jul 18;16(7):1053. doi: 10.3390/biom16071053 (PMC13406372; doi:10.3390/biom16071053)
Supplement: Supplementary file 1 [file biomolecules-16-01053-s001.zip › biomolecules-4282443-supplementary.pdf]

Supplemental Materials

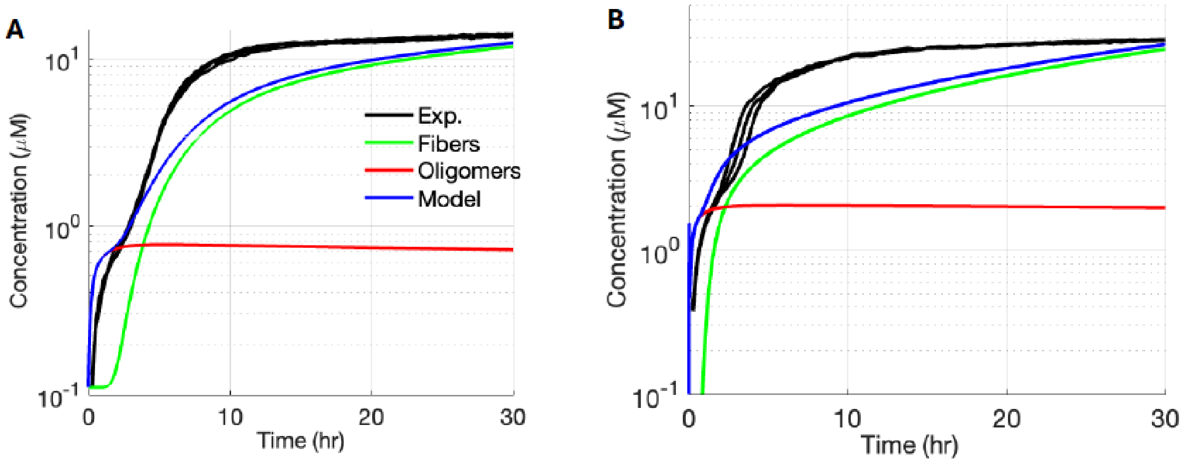

Figure S1: Biphasic Aβ42 aggregation kinetics at higher monomer concentration along with model fits. Traces at 15 μM (A) and 30 μM (B) where the black, red, green, and blue lines represent experimental data, off-pathway oligomers, on-pathway RFs, and the overall aggregated species given by the model, respectively. Experiment at each monomer concentration was repeated three times as shown by the three black lines in each panel.
